# Effects of nutrient metabolism on pancreatic β‐cell mass and function: Recent findings

**DOI:** 10.1111/jdi.14052

**Published:** 2023-07-09

**Authors:** Takuma Yasuda, Norio Harada

**Affiliations:** ^1^ Department of Diabetes, Endocrinology and Nutrition, Graduate School of Medicine Kyoto University Kyoto Japan

## Abstract

This article summarizes recent findings on the effects of nutrients on pancreatic ß‐cell mass and function. Further studies are expected to facilitate the prevention of the onset and treatment of diabetes by nutritional therapy.

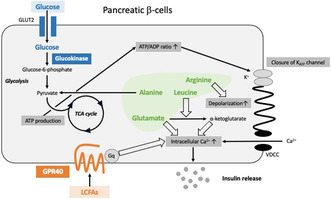

Insulin secretion from pancreatic β‐cells is required for glycemic control and is determined by both β‐cell mass and function. Decreased proliferation and function of β‐cells during adulthood leads to a higher risk of diabetes. Nutrient ingestion generally increases plasma insulin levels to preserve postprandial glucose homeostasis; glucose intake rapidly and markedly increases plasma insulin levels, while protein and fat intake increase plasma insulin levels gradually^1^, but excess exposure can lead to β‐cell dysfunction, loss, and diabetes. In fact, the metabolism of ingested nutrients can affect both β‐cell proliferation and function. Recently, several novel mechanisms have been elucidated.

While most adult β‐cells are well‐differentiated and are in a quiescent phase of the cell cycle (G0 phase), recent studies indicate that glucose ingestion regulates the expression levels of genes associated with cell cycle progression^2^, ^3^. Indeed, glucose activates the mitogenic transcriptional factor forkhead box M1 (FoxM1), which promotes G1/S transition through several cyclins to maintain β‐cell mass during adulthood^4^, ^5^. FoxM1 also contributes to the preservation of β‐cell mass by increasing the expression of transcription factor E2F1 and several mitosis‐related genes such as centromere protein A (CENP‐A) and polo‐like kinase 1 (PLK1)^2^, ^4^. In addition, E2F1 modulates insulin secretion by the transcriptional regulation of Kir6.2, a K_ATP_ channel component^6^. Moreover, β‐cell‐specific FoxM1‐deficient mice show reduced β‐cell mass and impaired insulin secretion compared with control mice, which leads to exacerbation of glucose intolerance^4^. Furthermore, glucokinase, a rate‐limiting enzyme for glycolysis, is involved in both insulin secretion and proliferation of β‐cells. Intracellular signaling for insulin secretion is initiated by influx of glucose into β‐cells and subsequent adenosine triphosphate (ATP) production through the glycolytic pathway and the tricarboxylic acid (TCA) cycle^7^ (Figure 1). Glucokinase promotes ATP production and induces the closure of K_ATP_ channels, which depolarizes the cellular membrane and opens the voltage‐dependent calcium channels (VDCC). The increased intracellular calcium enhances insulin granule exocytosis. Moreover, glucokinase stimulates β‐cell proliferation in response to exacerbated insulin resistance. A previous study reported that while β‐cell mass of wild‐type (Gck^+/+^) mice is increased by a high‐starch diet (HSTD) compared with that by control diet, the β‐cell mass of glucokinase‐haploinsufficient (Gck^+/−^) mice under HSTD did not differ from that of control diet‐fed Gck^+/−^ mice^8^. Thus, it is likely that glucokinase is required for the increase in β‐cell mass induced by HSTD feeding.

**Figure 1:**
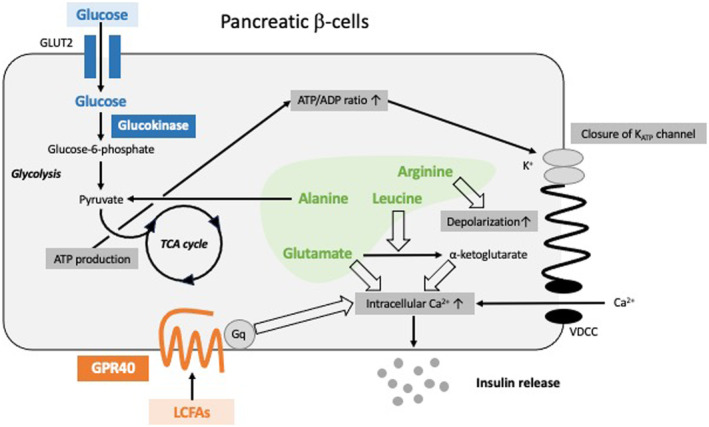
Nutrients related to insulin secretion from pancreatic β‐cells. The influx of glucose into β‐cells increases adenosine triphosphate (ATP) production through the glycolytic pathway and the tricarboxylic acid (TCA) cycle, which is promoted by glucokinase. Of the amino acids, alanine is converted to pyruvate and stimulates insulin secretion through the generation of ATP in the TCA cycle. Arginine, a positively charged amino acid, induces depolarization of the plasma membrane. In addition, while glutamate increases intracellular calcium levels, the conversion of glutamate to α‐ketoglutarate is more potent in insulin secretion than glutamate itself when activated by leucine. On the other hand, long‐chain free fatty acids (LCFAs) bind to the G protein‐coupled receptor 40 (GPR40), which increases intracellular Ca^2+^ levels. ADP, adenosine diphosphate; GLUT2, glucose transporter type 2; VDCC, voltage‐dependent calcium channel.

Amino acid metabolism is also strongly associated with insulin secretion from β‐cells through distinct pathways. While alanine is converted to pyruvate and stimulates insulin secretion through the generation of ATP in the TCA cycle, arginine, a positively charged amino acid, induces depolarization of the plasma membrane directly^9^, ^10^ (Figure 1). In a recent report, glutamate converted from glutamine was shown to promote calcium signaling at a high glucose concentration^11^. Moreover, conversion of glutamate to α‐ketoglutarate resulted in more robust insulin secretion than that by glutamate itself when activated by leucine. These findings suggest that glutamine supplementation might be able to augment insulin secretion. In addition, another report has shown that plasma concentrations of glutamate in patients with type 1 diabetes are significantly lower than those in healthy participants and in patients with type 2 diabetes^12^. Because the conversion of glutamate to α‐ketoglutarate by α‐ketoglutarate dehydrogenase is suppressed by insulin, insulin deficiency in type 1 diabetes might exacerbate the excessive glutamate metabolism. Indeed, the resultant excessive amino acid metabolism could lead to excessive hepatic glucose production by amino acids and subsequent hyperglycemia. However, the detailed mechanism of the decrease in plasma glutamate is not known. Further studies are required to clarify the mechanisms involved in amino acid induction of insulin secretion. Additionally, while there are few reports on the effects of amino acids on β‐cell proliferation, leucine has been shown to activate the mammalian target of rapamycin complex 1 (mTORC1) *via* the system‐L amino acid transporter 1 (LAT1), an active amino acid transporter of branched‐chain amino acids (BCAAs) and aromatic amino acids^13^. However, whether the change in amino acid levels affects β‐cell proliferation is not known.

Lipid metabolism also has associations with β‐cell proliferation as well as insulin secretion. Several G protein‐coupled receptors (GPRs) activated by fatty acids have been identified^14^, ^15^. GPR40/free fatty acid receptor 1 (FFAR1), a receptor for long‐chain free fatty acids (LCFAs), is expressed in β‐cells. GPR40 couples mainly with Gq protein and promotes Ca^2+^ release from endoplasmic reticulum (ER), which increases intracellular Ca^2+^ levels and potentiates glucose‐stimulated insulin secretion (Figure 1). On the other hand, excessive LCFAs secreted from ectopic fat in obese patients can cause lipotoxicity, which reduces insulin secretion from β‐cells^16^, ^17^. It has also been reported that saturated fatty acids promote ER stress, leading to apoptosis of β‐cells *via* unfolded protein response^18^. Moreover, adipose tissue in obese patients is contaminated with environmental pollutants such as organochlorine pesticides, polychlorinated biphenyls, dioxins, and polybrominated diphenyl ethers, which exacerbate hyperglycemia by impairing oxidative phosphorylation of mitochondria^19^. In addition, hypertriglycemia and hypercholesterolemia increase the risk of β‐cell dysfunction. In an oral fat tolerance test in participants with normal glucose tolerance, the fasting and postprandial triglyceride concentrations were found to be negatively correlated with the disposition index, which quantifies β‐cell function relative to insulin resistance^20^. Thus, both fasting triglyceride and postprandial triglyceride may be useful for the detection of the early stages of β‐cell dysfunction. Moreover, Goto‐Kakizaki (GK) rat normoglycemic/hypercholesterolemic neonates show islet microangiopathy, disturbed angiogenesis, collapsed vascularization, and reduced β‐cell mass^21^. It is therefore possible that deficient angiogenesis due to hypercholesterolemia can inhibit the cell cycle progression of β‐cells.

This article summarizes recent findings on the effects of nutrients on pancreatic β‐cell mass and function. Further studies are expected to facilitate the prevention of onset and treatment of diabetes by nutritional therapy.

## DISCLOSURE

N. H. received scholarship grants from Mitsubishi Tanabe Pharma Co., Ltd, Ono Pharmaceutical Co. Ltd, and Sanofi K.K. T. Y. declares no conflicts of interest.
